# Comparative Myocardial Deformation in 3 Myocardial Layers in Mice by Speckle Tracking Echocardiography

**DOI:** 10.1155/2015/148501

**Published:** 2015-03-02

**Authors:** Nicole Tee, Yacui Gu, Murni, Winston Shim

**Affiliations:** ^1^National Heart Research Institute Singapore, National Heart Centre Singapore, Singapore 169609; ^2^Duke-NUS, Graduate Medical School, Singapore 169857

## Abstract

*Background*. Speckle tracking echocardiography (STE) using dedicated high-resolution ultrasound is a relatively new technique that is useful in assessing myocardial deformation in 3 myocardial layers in small animals. However, comparative studies of STE parameters acquired from murine are limited. *Methods*. A high-resolution rodent ultrasound machine (VSI Vevo 2100) and a clinically validated ultrasound machine (GE Vivid 7) were used to consecutively acquire echocardiography images from standardized parasternal long axis and short axis at midpapillary muscle level from 13 BALB/c mice. Speckle tracking strain (longitudinal, circumferential, and radial) from endocardial, myocardial, and epicardial layers was analyzed using vendor-specific offline analysis software. *Results*. Intersystem differences were not statistically significant in the global peak longitudinal strain (−16.8 ± 1.7% versus −18.7 ± 3.1%) and radial strain (46.8 ± 14.2% versus 41.0 ± 9.5%), except in the global peak circumferential strain (−16.9 ± 3.1% versus 27.0 ± 5.2%, *P* < 0.05). This was corroborated by Bland Altman analysis that revealed a weak agreement in circumferential strain (mean bias ± 1.96 SD of −10.12 ± 6.06%) between endocardium and midmyocardium. However, a good agreement was observed in longitudinal strain between midmyocardium/endocardium (mean bias ± 1.96 SD of −1.88 ± 3.93%) and between midmyocardium/epicardium (mean bias ± 1.96 SD of 3.63 ± 3.91%). Radial strain (mean bias ± 1.96 SD of −5.84 ± 17.70%) had wide limits of agreement between the two systems that indicated an increased variability. *Conclusions*. Our study shows that there is good reproducibility and agreement in longitudinal deformation of the 3 myocardial layers between the two ultrasound systems. Directional deformation gradients at endocardium, myocardium, and epicardium observed in mice were consistent to those reported in human subjects, thus attesting the clinical relevance of STE findings in murine cardiovascular disease models.

## 1. Introduction 

Two-dimensional (2D) speckle tracking echocardiography (STE) has improved quantification of wall motion deformation in assessing cardiac performance. The STE technique captures myocardial features in greyscale *B*-mode images from interference of reflected ultrasound beam and presents them as unique speckle patterns [1]. Postprocessing of the speckle patterns through user-defined region of interest in image pixels and tracking their movement enable extraction of spatial and temporal data. These yield useful regional velocity, displacement, strain, and strain rate along the longitudinal, radial, and circumferential axes of the left ventricle. The STE technique has an advantage over tissue Doppler imaging (TDI) in angle independent assessments [2] and it is known to be highly reproducible when compared to 2D TDI and 3D TDI in clinical imaging [3].

STE is gaining clinical importance due to compelling validation against data gathered from magnetic resonance imaging (MRI), TDI, and sonomicrometry techniques in animal models [4–7] and in clinical settings [8–10]. However, it is recognized that different vendors employed disparate speckle tracking algorithms that are largely proprietary and comparative studies of different STE systems have not been extensively reviewed [11, 12]. Efforts by the American Society of Echocardiography (ASE) and the European Association of Echocardiography (EAE) to standardize analytical software have had limited success [1]. The usefulness of STE in identifying segmental LV dysfunction in mouse heart failure model has been demonstrated previously using a conventional clinical echocardiography system [13] and a high-resolution rodent ultrasound system [14]. However, cross-comparability of data acquired by the two ultrasound systems is unclear. Therefore, we sought to examine data consistency of 2D speckle-derived myocardial strain data captured and analyzed by a dedicated rodent Vevo 2100 system with a clinically validated Vivid 7 system to verify clinical relevance of our experimental findings in healthy mice.

## 2. Methods

### 2.1. Animal Preparation

All animal studies conducted were approved by Institutional Animal Care and Use Committees. A total of 13 male BALB/c mice were used. Mice were anesthetized at 2% isoflurane with 1 L/hr oxygen during induction for 20 minutes and were maintained at 1% to 1.5% isoflurane during imaging. Mice were fixed in the supine position on a heated platform with paws secured to the electrode pads covered with conducting gel for ECG monitoring when scanning with Vevo 2100 (VisualSonics, VSI, Toronto, Canada) system. ECG electrodes were placed onto the left and right limbs and left upper extremity of the mice when scanning with GE Vivid 7 (GE Healthcare, Horten, Norway). All heart rates (HR) were monitored and maintained at the average of 360–460 bpm. Hair removal cream was applied onto the chest, neckline, and upper and lower extremities of the mice. Care was taken to avoid excessive pressure while acquiring images, which was known to induce bradycardia.

### 2.2. Echocardiographic Image Acquisition

**Figure 1:**
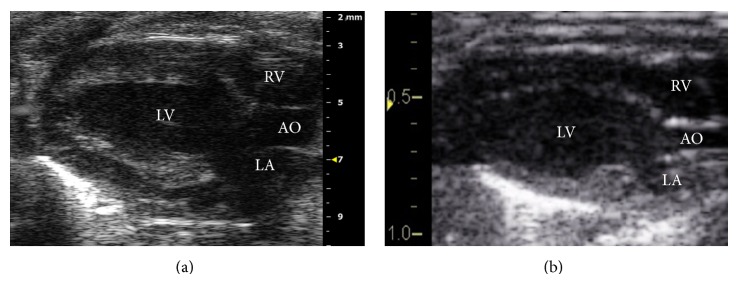
2D greyscale of parasternal long axis acquired by (a) VSI Vevo 2100 and (b) GE Vivid 7. LV: left ventricle; RV: right ventricle; LA: left atrium; AO: aorta.

**Figure 2:**
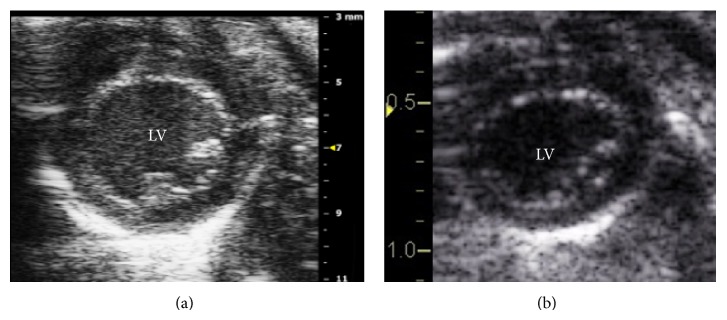
2D greyscale of parasternal short axis acquired by (a) VSI Vevo 2100 and (b) GE Vivid 7. LV: left ventricle.

Echocardiography was performed on GE Vivid 7 with i13L linear array transducer and Vevo 2100 with MS400 linear array transducer (Table 1). To ensure reproducibility, segments or images with acoustic shadowing or reverberations were omitted from the study. Special care was taken to optimize sector width for complete myocardial visualization while artifacts that resemble speckles influencing tracking quality were precluded. Gain settings were adjusted to optimize endocardial definition. Extra care was taken to maintain high frame rate to circumvent shifting of speckles in sequential frames without compromising imaging quality associated with reduced number of ultrasound beams in each frame in sustaining high frame rates [15]. Standard parasternal long axis (Figure 1) and short axis at midpapillary muscle level (Figure 2) views with frame rate more than 200 frames/sec with Vevo 2100 were obtained as per vendor recommendation for optimal speckle tracking analysis. Frame rate of 130–190 frames/sec was obtained with Vivid 7. Foreshortening view was omitted as it tended to underestimate true strain, thus affecting 2D STE results [15]. 2D guided *M*-mode of parasternal short axis at papillary muscle level was acquired to measure LV conventional parameters. Average of 10 cardiac cycles at each plane was stored in cineloop with both systems for subsequent offline analysis.

### 2.3. Postprocessing Analysis

2D STE applied myocardial lagrangian strain by following movement of stable patterns of acoustic markers frame by frame throughout the cardiac cycle. The shift of these acoustic markers represented tissue movement and provided spatial and temporal data used to calculate changes in length of the myocardium with the use of vendor-specific analysis software. Global peak radial (RS) and circumferential strains (CS) sampled from anterior, lateral, posterior, inferior, posteroseptal, and anteroseptal segments were measured from the short axis view. Clinically, global peak longitudinal strain (LS) is measured from apical 4-chamber view; however due to the anatomical position of rodent heart, apical 4-chamber view was not feasible; instead global peak LS was measured from anterior basal, mid, and apical and posterior basal, mid, and apical segments of long axis view. Midmyocardium strain data of GE images were analyzed by 2D-strain EchoPAC PC version 103.0.1 (GE Healthcare, Horten, Norway) while strain data from epicardial and endocardial segments of Vevo images were analyzed by VevoStrain version 1.3.0. (Visual Sonics, VSI, Toronto, Canada). The endocardial border was manually traced at the end systolic frame by point and click approach. Epicardial border was assimilated by the software automatically and was verified and accepted for analysis when no further adjustments were required. Segments with inadequate tracing were excluded from analysis.

2D guided *M*-mode of parasternal short axis was used to measure end diastolic diameter (LVEDD) and end systolic diameter (LVESD). Ejection fraction was calculated as LVEF (%) = [(LVEDD − LVESD)^2^/LVEDD^2^] × 100. Fractional shortening was calculated as FS (%) = [(LVEDD − LVESD)/LVEDD] × 100. All image acquisitions and offline measurements were conducted by an experienced sonographer. Unlike EchoPAC, VevoStrain does not display aortic valve closure (AVC) and is not as apparent in detecting delay in LV contraction. However, the AVC displayed on strain measurement is based on the AVC marked down in left ventricle outflow tract (LVOT) pulse wave Doppler in EchoPAC. Therefore, heart rate during the Doppler analysis might differ during the strain analysis. In such case, the AVC timing has to be corrected by heart rate through the following formula: AVC_*s*_ = AVC_*d*_ × (*R* − *R*_*s*_/*R* − *R*_*d*_)^1/2^, where *R* − *R*_*s*_ interval derived from 2D strain and *R* − *R*_*d*_ and AVC_*d*_ derived from Doppler tracing. AVC_*s*_ is aortic valve closure time in 2D strain.

### 2.4. Statistical Analysis

Data were presented as mean ± standard deviation. Paired *t*-test was used to detect any significant difference in conventional echo parameters and strain measurements. *P* values < 0.05 were considered statistically significant. Global peak strain was calculated as the average of all measurable segments. The mean difference and limits of agreement (95% confidence interval) between measurements derived from each system were calculated. Agreement between systems was determined by Bland Altman analysis [16]. Five randomly selected mice were reanalyzed for their global peaks LS, CS, RS, and LVEF by a second sonographer to determine interobserver variability. Interobserver agreement was calculated using intraclass correlation coefficient [17]. Statistical analysis was performed using SPSS software (version 20.0, SPSS Inc., Chicago, IL, USA).

## 3. Results

A total of 78 segments were acquired (6 segments each view × 13 animals). Midmyocardium LS (−16.8 ± 1.7%; 74/78 segments analyzed) by EchoPAC was not statistically different as compared to endocardial LS (−18.7 ± 3.1%, *P* = 0.11, 76/78 segments analyzed), but it was significantly different from those in epicardial LS (−13.2 ± 4.3%, *P* < 0.05, 70/78 segments analyzed) by VevoStrain (Table 3).

Midmyocardium CS (−16.9 ± 3.1%, 74/78 segments analysed) by EchoPAC differed significantly from endocardial CS (−27.0 ± 5.2%, *P* < 0.05, 77/78 segments analyzed) and epicardial CS (−11.3 ± 2.0%, *P* < 0.05, 76/78 segments analyzed) measured using VevoStrain (Table 3). Peak RS did not differ significantly between EchoPAC (46.8 ± 14.2%, *P* = 0.26, 75/78 segments analysed) and VevoStrain (41.0 ± 9.5%, 76/78 segments analyzed) (Table 3). No significant differences were found in mean HR computed by EchoPAC or VevoStrain (*P* = 0.245). Similarly, LVEF (*P* = 0.13) and FS (*P* = 0.11) from *M*-mode analysis were not significantly different between the two systems (Table 2).

**Figure 3:**
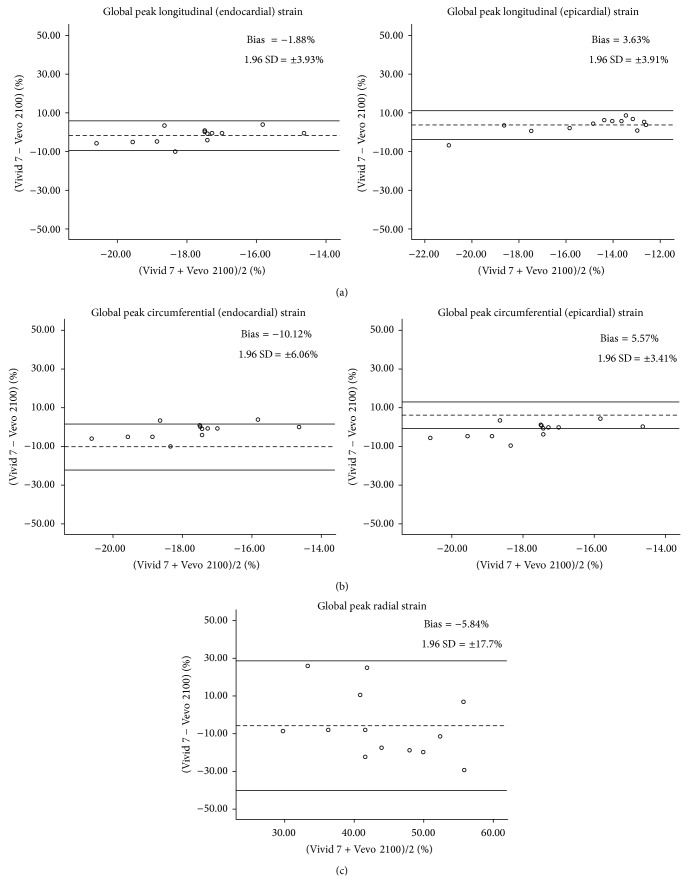
Bland Altman plot depicts the agreement of strain analysis between EchoPAC (Vivid 7) and VevoStrain (Vevo 2100). (a) Global peak longitudinal strain between midmyocardium/endocardium and midmyocardium/epicardium shows narrower variation than (b) global peak circumferential strain and (c) global peak radial strain. Dotted horizontal lines denote bias (mean difference between two systems) and solid horizontal lines illustrate the 95% limits of agreement.

Analysis by scatter diagrams and Bland Altman plots showed that global peak LS had a better agreement than global peak CS and RS between the two systems (Figure 3). The mean bias and limits of agreement (1.96 SD) between endocardial LS and midmyocardium LS was smallest at −1.88 ± 3.93% and followed by between epicardial LS and midmyocardium LS at 3.63 ± 3.91%. The peak CS was identified to have a major bias with mean bias ± 1.96 SD of −10.12 ± 6.06% and 5.57 ± 3.41% at endocardial and epicardial segments, respectively. Mean bias in peak RS was found to have the widest limits of agreement with mean difference of −5.84 ± 17.70% (Figure 3).

The variability of global peaks LS, CS, and RS measurements using EchoPAC between two independent observers was highly reproducible with intraclass correlation coefficient at 1.0, 0.79, and 0.94, respectively. The variability of global peak LS for endocardium and epicardium analyzed using VevoStrain showed good correlation at 0.97 and poorer one at 0.55, respectively. While variability of global peak CS for endocardium and epicardium was 0.93 and 0.54, respectively, variability of global peak RS measured was 0.93.

## 4. Discussion

Mouse represents a critical model in the understanding of LV dysfunction in cardiovascular diseases. Changes to LV structure and function detected by conventional echocardiographic parameters, such as fractional shortening (FS) or ejection fraction (EF), are considered to be late manifestation of disease. In contrast, STE and speckle tracking-based strain analysis are found to provide greater sensitivity and specificity in detecting subtle early changes of cardiac performance in cardiac pathophysiology.

There are increasing compelling data validations of 2D STE against data from MRI, tissue doppler imaging, and sonomicrometry in animal models and clinical studies in detecting abnormal LV function are emerging [4, 5]. Nevertheless, considerable challenges remain in STE imaging of rodents due to their small size, heart orientation, and rapid heart rates. High-resolution rodent ultrasound systems have been introduced to circumvent such limitations in STE and strain analysis [6, 14, 18]. It is assumed that strain calculation of the relative change in length between individual speckle by formula (*ε*) = (*L*_1_ − *L*_0_)/*L*_0_, whereby *L*_0_ is the original length. However, it is recognized that different systems employed disparate speckle tracking algorithms that are largely proprietary with unknown cross comparability. Comparative data between different STE systems have not been extensively reported [11, 12].

We observed in this study that there was a better agreement of LS measurements between the two systems than the CS and RS measurements by Bland Altman plots. Furthermore, better agreement was shown in longitudinal strain between midmyocardium and endocardium (mean bias of −1.88%; 1.96 SD of ±3.93%) than with epicardium (mean bias of 3.63%; 1.96 SD of ±3.91%). This coincided with clinically observed parallel gradient for longitudinal strain between endocardial and midmyocardial layers, but not epicardium and midmyocardium [19], which supported the layer-specific statistical differences observed in strain values (Table 3). The weak agreement observed (−10.12%) in the Bland Altman plot of circumferential strain between endocardium and midmyocardium may be accentuated by differential muscle fiber orientation between the midmyocardial layer (mainly circumferential) and its two adjacent layers (mainly longitudinal) that affects myocardial layer deformation characteristics [12].

It is well recognized that LS and CS are the highest in endocardium followed by myocardium and epicardium in healthy human subjects, though discrepancy has also been noted [19, 20]. Consistently, our study in mice similarly found that VevoStrain's endocardial LS produced the highest value (−18.7 ± 3.1%), and epicardial LS recorded the lowest value (−13.2 ± 4.3%) while EchoPAC's midmyocardium LS reported an intermediate value (−16.8 ± 1.7%) that is in agreement with the “averaging effect” in EchoPAC, where values generated represent the mean of all three cardiac layers [12]. Similar concordance results on CS values were recorded, whereby VevoStrain endocardial strain reported a higher CS value (27.0 ± 5.2%) than that of EchoPAC's midmyocardial strain (−16.9 ± 3.1%), while epicardial strain displayed the lowest CS value (11.3 ± 2.0%). These findings reaffirmed the clinical relevance of STE from preclinical experimental models.

**Figure 4:**
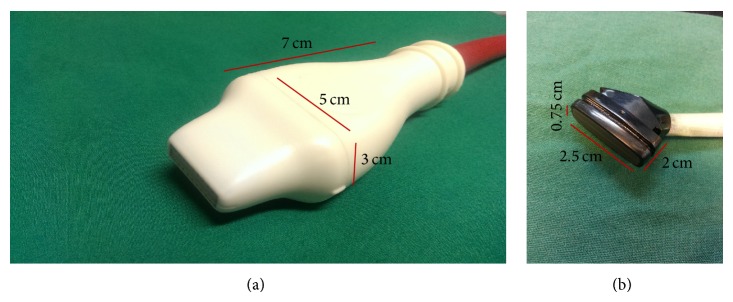
VSI MS 400 transducer (a) with frequency of 18–38 MHz. GE i13L transducer (b) with frequency of 10–14 MHz.

**Figure 5:**
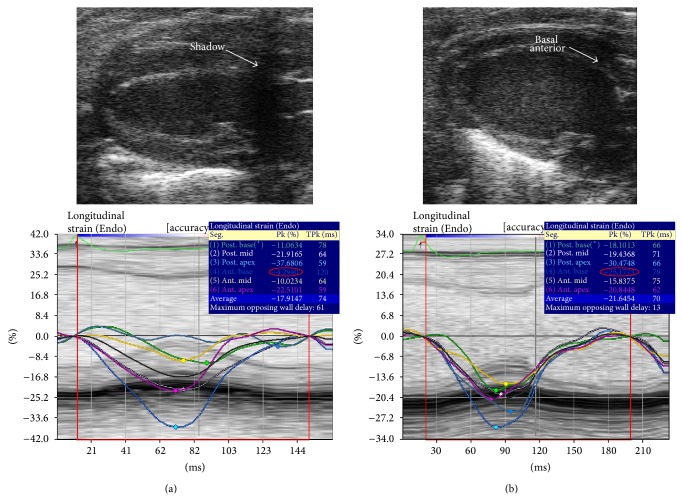
Representation of 2D parasternal long axis where (a) showed basal anterior obscured by shadowing due to sternum but with slight adjustment; shadowing can be eliminated (b). Speckle tracking (below) showed a significant difference in the reading.

Due to different design of the two transducers (Figure 4), i13L of the GE system offers a more effortless and flexible imaging, though the MS400 of Vevo 2100 affords an additional option of hand-free handling. Similar to percentage excluded segments reported previously [12], about 11% of the excluded LS segments analyzed using Vevo 2100 were basal anterior segment where the images were obscured or affected by shadows from sternum (Figure 5(a)). We minimized such obstruction by adjusting transducer angle, but at the expense of tilting of left ventricle apex (Figure 5(b)). However, this did not affect data integrity as STE has the advantage of angle independence as compared to TDI. The Vevo 2100 machine provides animal handling and physiological monitoring system that tracks not only heart rate more than 300 beats per minute, but also animal's respiratory cycle and temperature. We found it most useful in choosing good cardiac cycle during expiration of the respiratory cycle for analysis as cardiac strain analysis during inspiration invariably produced poorer results.

**Figure 6:**
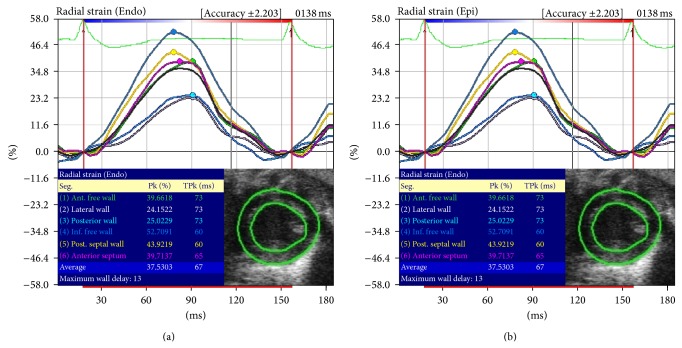
Representation of VSI VevoStrain endocardial (b) and epicardial (a) radial strain showing the same value.

Radial strain (RS) values from VevoStrain were derived from taking corresponding points at the epicardium and endocardium and averaging them across the radial distance between the two points (Figure 6). Our study did not reveal significant RS differences between our two ultrasound systems. However, we found that RS has a wide limit of agreement by Bland Altman plot that indicated greater variability (Figure 3), which is in concordance with previous reports [2, 11, 12, 21, 22].

Lack of reproducibility has been reported as a major drawback of 2D STE, especially in RS analysis [12, 21–23]. However, there was good reproducibility in our interobserver test for variability in the measurements of LS, CS, and RS on both systems. Nevertheless, the exact reason for greater variability experienced in RS analysis is uncertain and was not apparent from our study. It is presumed that inadequate tracking of the epicardial border and respiratory-related lung artefacts (especially in the antero/lateral segments) play a major contributing role [22].

## 5. Limitations

LV borders were traced semiautomatically by both systems during strain analysis; the two systems are however designed to analyze different cardiac muscle layers. EchoPAC analyzes midmyocardium while VevoStrain analyzes endocardium and epicardium that may inadvertently introduce variability in comparison. Furthermore, it is noted that, without flexibility to adjust individual point, the ROI width remains fixed around the whole circumference in EchoPAC; thus overestimation or underestimation was likely to have occurred during strain analysis. Lastly, our current observations were derived from healthy mice; it will be interesting to ascertain if similar differences between the two systems are replicated in diseased animal models. Nevertheless, similar changes in radial strain recorded with Vivid 7 ([13]; as reported in Figure 2) and Vevo 2100 ([14]; as reported in Table 3) ultrasound systems were separately observed in C57/B6 mice with LV dysfunction by 2 independent groups previously.

## 6. Conclusions

Our study showed that there is a good agreement in LS in the 3 myocardial layers between Vevo 2100 and Vivid 7 ultrasound systems which lends credence to validity of findings from experimental models. Our strain analysis showed that, similar to healthy human subjects [19], there is a gradient of contractile deformation from endocardium towards epicardium in mouse model that may be useful for detailing early changes in myocardial performance in a muscle layer-centric and cardiac segment-specific manner for monitoring disease progression in pathophysiological conditions.

## Figures and Tables

**Table 1:** Comparison between GE Vivid 7 and Vevo 2100 in 2D-speckle tracking hardware and software abilities.

Hardware abilities		
	GE Vivid 7	Vevo 2100
Transducer frequency	10–14 MHz	18–38 MHz
Axial resolution	200 *µ*m	50 *µ*m
Lateral resolution	300 *µ*m	110 *µ*m
Transducer footprint	28 mm × 10 mm	20 mm × 5 mm
Temporal resolution	130–190 fps	>200 fps
Detect respiratory cycle	Yes	Yes
Image acquisition	Conventional	Hand-free + conventional
Area of analysis	Myocardium	Endocardium + epicardium
ROI adjustment	Uniform	Individual
Ability to distinguish E/A wave when heart rate is more than 400 bpm	No	Yes
Marked AVC	Yes	No
FAC	Manual	Automated

fps: frame per second, AVC: aortic valve closure, and FAC: fractional area change (defined as cross-sectional area change between end diastole and end systole).

**Table 2:** Consecutively acquired GE Vivid 7 and Vevo 2100 conventional parameters.

	GE EchoPAC	VSI VevoStrain	*P* value	95% CI
Mean HR (bpm)	401 ± 82	384 ± 46	NS	−67.2 to 33.2
Mean LVEF (%)	59.6 ± 7.5	57.6 ± 7.3	NS	−0.27 to 4.2
Mean FS (%)	36.7 ± 6.1	35.1 ± 5.9	NS	−0.11 to 3.3

**Table 3:** Consecutively acquired GE Vivid 7 and Vevo 2100 global peak strains.

	GE EchoPAC	VSI VevoStrain	*P* value	95% CI
Global peak longitudinal strain (%)	−16.8 ± 1.7	−18.7 ± 3.1 (Endo)	NS	−4.2 to 0.5
		−13.2 ± 4.3 (Epi)	0.006	1.3 to 6.0

Global peak circumferential strain (%)	−16.9 ± 3.1	−27.0 ± 5.2 (Endo)	<0.05	−13.8 to −6.5
		−11.3 ± 2.0 (Epi)	<0.05	3.5 to 7.6

Global peak radial strain (%)	46.8 ± 14.2	41.0 ± 9.5	NS	−16.5 to 4.9
